# Effects of temperature on the interaction between amphibian skin bacteria and *Batrachochytrium dendrobatidis*

**DOI:** 10.3389/fmicb.2023.1253482

**Published:** 2023-10-24

**Authors:** Matthew J. Robak, Veronica Saenz, Esmee de Cortie, Corinne L. Richards-Zawacki

**Affiliations:** ^1^Department of Ecology and Evolutionary Biology, Tulane University, New Orleans, LA, United States; ^2^Department of Biological Sciences, University of Pittsburgh, Pittsburgh, PA, United States; ^3^Department of Biology, The Pennsylvania State University, State College, PA, United States; ^4^Falk School of Sustainability and Environment, Chatham University, Pittsburgh, PA, United States

**Keywords:** Chytridiomycosis, antifungal bacteria, host-pathogen interactions, amphibian, microbiome

## Abstract

Symbiotic relationships between animals and microbes are important for a range of functions, from digestion to protection from pathogens. However, the impact of temperature variation on these animal-microbe interactions remains poorly understood. Amphibians have experienced population declines and even extinctions on a global scale due to chytridiomycosis, a disease caused by chytrid fungi in the genus *Batrachochytrium*. Variation in susceptibility to this disease exists within and among host species. While the mechanisms generating differences in host susceptibility remain elusive, differences in immune system components, as well as variation in host and environmental temperatures, have been associated with this variation. The symbiotic cutaneous bacteria of amphibians are another potential cause for variation in susceptibility to chytridiomycosis, with some bacterial species producing antifungal metabolites that prevent the growth of *Bd*. The growth of both *Bd* and bacteria are affected by temperature, and thus we hypothesized that amphibian skin bacteria may be more effective at preventing *Bd* growth at certain temperatures. To test this, we collected bacteria from the skins of frogs, harvested the metabolites they produced when grown at three different temperatures, and then grew *Bd* in the presence of those metabolites under those same three temperatures in a three-by-three fully crossed design. We found that both the temperature at which cutaneous bacteria were grown (and metabolites produced) as well as the temperature at which *Bd* is grown can impact the ability of cutaneous bacteria to inhibit the growth of *Bd*. While some bacterial isolates showed the ability to inhibit *Bd* growth across multiple temperature treatments, no isolate was found to be inhibitive across all combinations of bacterial incubation or *Bd* challenge temperatures, suggesting that temperature affects both the metabolites produced and the effectiveness of those metabolites against the *Bd* pathogen. These findings move us closer to a mechanistic understanding of why chytridiomycosis outbreaks and related amphibian declines are often limited to certain climates and seasons.

## 1. Introduction

The importance of symbiotic relationships between animals and microbes has long been appreciated thanks to research in a few well-studied systems, like coral reefs (Li, 2019), hydrothermal vents (Petersen et al., 2011), and the gut microbiomes of animals who specialize on difficult to digest and/or nutrient poor food sources (e.g., termites; Brune and Ohkuma, 2010). However, the recognition that all animals live in symbiotic association with bacteria and other microorganisms is a recent development, and one that is dramatically changing the field of biology (Chow et al., 2010). With this change in our scope of understanding of host-microbiome interactions comes a need to understand how the future of such interactions may be affected by environmental change (Carey and Duddleston, 2014). In many cases, we know little about how variation in temperature, a variable that shapes many aspects of the physiology and behavior of animal hosts, affects the services that microbial symbionts provide. A clearer understanding of temperature’s impact on such symbiotic relationships is needed to predict wildlife responses to climate change and other environmental stressors.

Effects of temperature on the growth and community structure of animal microbiomes has not been well studied. Much more is known about effects of temperature on microbial communities in soil and in other environmental samples. For example, bacterial diversity and community structure is thought to be primarily controlled by environmental temperature in permafrost and other low-temperature environments (Jansson and Taş, 2014). This predominant effect of temperature has also been observed in Arctic fjord soils, with productivity, abundance, and proportion of active bacterial cells increasing during the spring (Piquet et al., 2016). From the few studies available, most of which involve marine animals or insects, the pattern for animal microbiomes appears to be more varied. For example, in one study sponge-associated bacterial communities were found to remain stable across large seasonal shifts in temperature (between 12°C and 26°C; Erwin et al., 2012). Yet, when bacterial communities associated with the sponge *Rhopaloeides oborabile* were exposed to temperatures ranging from 27°C to 33°C, differences in community structure were observed (Webster et al., 2008). Furthermore, elevated temperatures in *R. oborabile* have been shown to cause an immediate stress response in both the host and its microbial community (Fan et al., 2013). Though examples are rarer, temperature appears to affect the bacterial communities of insects as well. For example, in an experimental evolution study using *Drosophila melanogaster* flies as hosts to several strains of *Wolbachia*, which strain spread in the fly population was found to depend upon the temperature at which the population was maintained (Versace et al., 2014).

In some animals, temperature is known to affect the potential of symbiotic microbes to aid their hosts in resisting infection or other forms of attack. However, the way in which temperature affects the host-enemy-symbiont interaction varies greatly among the systems in which it has been studied (reviewed in Corbin et al., 2017). For example, elevated temperatures have been shown to increase the expression of virulence genes in *Vibrio shiloi*, which causes bleaching in the coral *Oculina patagonica* (Rosenberg and Ben-Halm, 2002). On the other side of the coin, the defense of ascidians (sea squirts) against pathogens can be bolstered by diverse secondary metabolites produced by their microbial communities. Differences in the metabolites produced by the bacterial communities of these animals, and correspondingly the ability of these bacteria to protect the animal from infection, may be caused by changes in water temperature (Tianero et al., 2015). In insects the effects of temperature on the protective ability of microbes appear to vary. For example, temperature did not affect the production of antibiotics, which protect the cocoons of the European beewolf from attack by symbiotic *Streptomyces* (Koehler and Kaltenpoth, 2013). However, protection of pea aphids from attack by parasitic wasps, which is mediated by *Hamiltonella defensa*, was found to be thermally sensitive (Guay et al., 2009). Because they are ectothermic and threatened globally by a newly-emerged fungal pathogen, *Batrachochytrium dendrobatidis* (hereafter *Bd*), amphibians are another potentially fruitful animal study system for investigating the effects of temperature on microbial symbioses.

The symbiotic bacteria on amphibian skin are known to be important in defense against the pathogenic fungus *Batrachochytrium dendrobatidis* (hereafter *Bd*; Harris et al., 2009; Woodhams et al., 2015), which causes the disease chytridiomycosis that has been linked to declines and even extinctions of amphibian species on several continents (Berger et al., 1998; Martel et al., 2013; Scheele et al., 2020). *Bd* is a microscopic chytrid fungus that attacks amphibian skin, feeding on keratin and producing motile zoospores that can be transmitted by contact between infected individuals or through contaminated water or substrate (Longcore et al., 1999; Kolby et al., 2015). Host mortality occurs when infections cause an imbalance of ions across amphibian skin resulting in cardiac arrest (Voyles et al., 2009). *Bd*-related population declines have been correlated with differences in climate and the seasonality of temperature and humidity (Berger et al., 2004; Kriger and Hero, 2006, 2007; Bishop et al., 2009; Rohr and Raffel, 2010), with declines and extinctions of amphibian hosts more likely to occur in cooler, wetter, less thermally variable areas (Berger et al., 2016).

Variation in susceptibility to chytridiomycosis exists both within and among amphibian species (Tobler and Schmidt, 2010; Martel et al., 2014) and may be caused by several different factors. Differences in virulence among strains of *Bd* appear to contribute to the global pattern of chytridiomycosis-related declines (O’Hanlon et al., 2018; Byrne et al., 2019). *Bd* strains can differ in their pathogenicity (Carey et al., 2006; Retallick and Miera, 2007), however, differences in virulence may also come from the host side of this interaction. For example, amphibian host species differ dramatically in the pathogen loads required to trigger clinical symptoms of disease (i.e., pathogen tolerance; Berger et al., 2004; Reeder et al., 2012; Rollins-Smith, 2020).

Amphibians have well-developed immune systems with several defenses that can be effective in combating *Bd*. One important component of the innate amphibian defense against chytridiomycosis is the secretion of antimicrobial peptides (AMPs), which are synthesized in glands in the skin of amphibians and secreted into the mucus. AMPs, part of the innate immune response, have been shown to inhibit the growth of *Bd* (Rollins-Smith et al., 2006; Ramsey et al., 2010); however, not all amphibian species produce AMPs and not all AMPs inhibit *Bd* growth (Conlon, 2011). Amphibians can also deposit antibodies in their mucus, which may contribute to defense against *Bd* (Ramsey et al., 2010). Acquired immunological resistance to *Bd* has also been observed (McMahon et al., 2014). However, *Bd* can also fight back and suppress the acquired immune system by releasing factors that inhibit lymphocyte responses both *in vitro* and *in vivo* (Fites et al., 2014; Rollins-Smith et al., 2015).

The microbiome on amphibian skin also appears to be important in defense against *Bd* (Harris et al., 2006, 2009; Flechas et al., 2012; Bell et al., 2013). The mucus on amphibian skin harbors a diverse community of microbes, and evidence suggests that these microbial communities tend to be species-specific (McKenzie et al., 2012). It has been suggested that AMPs may even play a secondary, supplemental role to bacteria in amphibians’ defense systems (Conlon, 2011), but they can also work synergistically with inhibitory bacteria (Myers et al., 2012). The presence of anti-*Bd* bacteria in skin mucus can increase host survival after infection with *Bd*, and this effect appears to be mediated by bacterial production of antifungal metabolites (Harris et al., 2009). For example, violacein, a substance produced by the bacterium *Janthinobacterium lividum*, has been shown to inhibit the growth of *Bd* (Becker et al., 2009; Harris et al., 2009). In some cases, a few individual bacterial strains within the cutaneous microbiomes of some amphibian hosts may exhibit broad-spectrum inhibition of *Bd* growth, but the bacterial community composition as a whole can also be a predictor of an animal’s ability to survive infection (Antwis et al., 2015; Becker et al., 2015a,b; Woodhams et al., 2015). This may be due to additive and synergistic effects of multiple bacterial strains within the community (Loudon et al., 2014), although it has also been noted that *Bd* infection can alter cutaneous bacterial communities as well (Jani and Briggs, 2014).

The optimal environmental conditions for antifungal metabolite production by beneficial amphibian skin bacteria are unknown, yet previous studies have found that temperature does play a role in the production of bacterial products in general (e.g., folate; Kariluoto et al., 2010). Temperature has also been shown to interact with the composition of the amphibian skin microbial community to impact the ability of amphibian hosts to tolerate *Bd* infections (Robak and Richards-Zawacki, 2018). If temperature can affect the production of key antifungal products by cutaneous bacteria, knowing this may help to explain the timing and locations of disease-related declines in amphibians. In areas and seasons, where environmental conditions are less conducive to the production of these antifungal products, amphibians may be more susceptible to *Bd* infection and chytridiomycosis and thus, in greater need of human-mediated disease management interventions.

Previous studies have demonstrated the presence of anti-*Bd* bacteria on amphibian skin, but few have addressed the effects of environmental variables on the ability of those bacteria to inhibit the growth of *Batrachochytrium*. Daskin et al. (2014) tested whether growth temperature affects the antifungal properties of cutaneous bacteria isolated from the skins of three species of rainforest tree frogs (*Litoria nannotis, L. rheocola,* and *L. serrata*). They found that bacteria grown under cooler temperatures produced fewer antifungal products and were less effective at inhibiting the growth of *Bd* than when these same bacteria were grown at higher temperatures; however, these products were only tested for their effectiveness against *Bd* at 23°C, a temperature at which *Bd* grows very well in culture (Piotrowski et al., 2004). If *Bd* was challenged to grow in the presence of these low-temperature bacterial products away from *Bd*’s optimal growth temperature (e.g., at the temperature lower temperature they were cultured in), their effectiveness against *Bd* may have been different.

The objective of the present study was to examine not only whether temperature affects the production of anti-*Bd* products by amphibian skin bacteria, but also to ask whether temperature modulates the effectiveness of these antifungal products against *Bd*. Specifically, we tested the hypotheses that (1) extracts from cutaneous bacteria cultured at different temperatures differ in their ability to inhibit *Bd* growth, perhaps due to differences in the types and/or quantities of metabolites produced, and (2) the effectiveness of these metabolite cocktails at inhibiting *Bd* growth depends on the temperature at which the interaction with *Bd* occurs (i.e., challenge temperature). We predicted that the amphibian skin bacteria would produce more metabolites with antifungal properties near their own optimal growth temperatures, which we predict may be more similar to the frogs’ typical body temperatures than to the optimal growth temperature for *Bd*. We also predicted that antifungal metabolites produced by these bacteria would be more effective at inhibiting *Bd* growth at temperatures that are suboptimal for *Bd* growth *in vitro* than at temperatures near 23°C, which is considered optimal for growth of *Bd* in culture (Piotrowski et al., 2004). To test these predictions, and to better our understanding of the dynamics and role of antifungal bacteria in this host-pathogen interaction, we collected cutaneous bacteria from two North American frog species with different mean body temperatures and patterns of habitat use in the wild: Blanchard’s cricket frogs (*Acris blanchardi*) and Southern leopard frogs (*Rana sphenocephala*). We grew the bacterial isolates from each species to a common optical density under three different temperatures and then challenged *Bd* to grow in the presence of their metabolic products at the same three temperatures (i.e., a three-by-three fully crossed experimental design).

## 2. Materials and methods

### 2.1. Bacteria collection and isolation

We captured five adult Blanchard’s cricket frogs (*Acris blanchardi*) from Tulane University’s F. Edward Hebert Riverside Research Center near Belle Chasse, Louisiana (29.8852489 °N, 89.9694904 °W) and five adult southern leopard frogs (*Rana sphenocephala*) from Payne’s Prairie near Gainesville, Florida (29.577939 °N, 82.312629 °W). We chose these species because they are both known to be susceptible to *Bd* but they differ in their habitat use and thermal ecology, making our findings both ecologically relevant and also potentially informative to conservation actions. *Acris blanchardi* is often found at the edge of ponds and slow-moving streams and tends to avoid wooded areas and dense vegetation (Hulse et al., 2001; Gamble et al., 2008). *Rana sphenocephala* can be found in all types of shallow freshwater habitats and will move into moist vegetation in terrestrial habitats to feed during the summer (Wright and Wright, 1949; Conant and Collins, 1991). Based on measurements made across seasons in Central Louisiana, *Acris blanchardi* (N = 330) have a mean body temperature of 22.5°C and a range of 8.9 to 27.9°C and *Rana sphenocephala* (N = 425) have a mean temperature of 19.5°C and a range of 8.6 to 30°C (M. Ohmer, unpublished data). We chose two harvest bacteria from two host taxa because skin microbial communities are known to vary greatly from species to species (McKenzie et al., 2012) and we hypothesized that differences in the patterns we see with temperature may arise from differences in the thermal ecology of the host species.

We captured frogs by hand, wearing clean nitrile gloves and changing them between each frog, and then rinsed each frog twice with dechlorinated water to remove transient bacteria. We collected symbiotic bacteria from frog skin by swabbing the frogs’ ventral and dorsal surfaces in the field; we swabbed five times on each surface, with a single dry, sterile swab (MW113, Medical Wire and Equipment Co. LTD.). This was repeated for a total of ten swabs per frog. After swabbing, we released all frogs at their point of capture. We then transported the swabs in 2 mL microcentrifuge tubes without any media, at ambient temperature, to a sterile environment where we streaked five swabs from each frog onto 1% tryptone agar plates and the other five swabs onto R2A agar plates. The total time from swabbing to streaking was 30–60 min. We then incubated each plate at 26°C until colonies could be isolated. We chose a total of 44 bacterial isolates for use in the challenge assays based on differences in morphology, with 22 coming from each frog species. Of the 22 per species, 11 came from the R2A plates and 11 from the 1% tryptone plates. We isolated these into pure culture using standard lab technique. The isolates chosen from the R2A plates were subsequently grown on 1% tryptone plates to ensure that they could be grown in the same broth medium as the other isolates during the challenge assays. We then grew each of the selected isolates in 1 mL of 1% tryptone broth medium for 24 h in two sterile cryotubes. Afterwards, we added 1 mL of glycerol to one tube, and placed the isolate in a − 20°C freezer to preserve it as a working stock and added 0.15 mL of glycerol to the other and placed the isolate in a − 80°C freezer as a preserved culture.

### 2.2. Bacterial identification

We identified bacterial isolates based on their 16S rRNA gene sequence. We extracted genomic DNA from each isolate using Qiagen’s DNeasy Blood and Tissue kit, following the protocol for gram-negative bacteria. This allowed for extractions from both gram-positive and gram-negative bacteria. We used primers 8F and 1492R (Turner et al., 1999) to amplify an approximately 1.5 kbp fragment of the 16 s rRNA gene. Each polymerase chain reaction (PCR) contained 40% GoTaq Green Master Mix (Promega), 10% 10uM 8F primer, 10% 10uM 1492R primer, 10% template (extracted genomic) DNA, and 30% molecular grade water by volume in a total reaction volume of 10 μL. The PCR was run for 4 min at 94°C followed by 30 cycles of 94°C for 1 min, 53°C for 1 min, and 72°C for 90 s; and then a final extension of 72°C for 10 min. We sent PCR products to Beckman Coulter Genomics for Sanger sequencing. Forward and reverse reads were aligned in Geneious Prime to create consensus sequences for each isolate, and these were then used in a standard nucleotide BLAST against NCBIs 16S rRNA sequences database, with default search parameters to identify the closest genus or species match, where available. We also used the usearch-global tool in VSEARCH (Rognes et al., 2016) to compare our sequences to those in the antifungal isolates database of amphibian skin-associated bacteria (Woodhams et al., 2015), which was last updated with additional culture data (7,340 isolates total) by MC Bletz and DC Woodhams in June of 2020.

### 2.3. Bacterial preparation for assays

To prepare the bacterial growth assay, we pipetted 200 μL of autoclaved 1% tryptone broth into each well of a sterile 96-well cell culture (Costar 3,595) plates. We then inoculated the wells of each plate with 15 different bacterial isolates, using six replicates of each isolate on each plate. This left six tryptone-only wells as blanks on each plate. We incubated replicate plates at 14°C, 20°C, and 26°C until isolates reached an optical density (OD) of 0.6 on a Spectramax 190 (Molecular Devices, LLC) spectrophotometer with a 492 nm filter. These temperatures were chosen because they are within the range where *Bd* grows well in culture and also within the range of body temperatures measured for wild individuals of our focal amphibian hosts. Upon reaching our target density, we pipetted isolates into 2 mL collection tubes and centrifuged at 9,000 rpm for 5 min to pellet cells. We then pipetted off the supernatant and pushed it through a syringe with a sterile 0.22 μm Millipore membrane filter, leaving the bacterial extracts in the filtered supernatant to be used in the *Bd* growth challenge assays. These extracts were stored at −20°C prior to use. We repeated this process for all 44 morphologically distinct isolates.

### 2.4. Challenge assays

We cultured *Bd* isolate JEL 412 (provided by Joyce Longcore, University of Maine), in its ninth passage, on TGhL plates at 23°C for use in the challenge assays. We chose JEL 412, which was isolated from a dying *Sachatamia ilex* frog in El Cope, Panama in 2005 during the chytridiomycosis epizootic there, because it was available at a low passage number, is known to retain its pathogenicity to amphibian hosts, and falls within the *Bd*GPL lineage (Byrne et al., 2019), which has been associated with declines and extinctions globally. We flushed *Bd* cultures in their seventh day of growth using TGhL broth and filtered them through a 20 μm filter to remove sporangia. We then resuspended the *Bd* to a working concentration of 1×10^6^ zoospores/ml. To test for inhibition of *Bd* growth by the bacterial products, we added 50 μL of each bacterial extract, obtained after growing the bacteria under the three temperatures, to three replicate wells of a 96 well culture plate (Costar 3,595). Then, we added 50 μL of *Bd* to each well containing the extracts. Each plate also had three positive control wells, consisting of 50 μL *Bd* and 50 μL of 1% tryptone broth, and three negative control wells, consisting of 50 μL heat-killed *Bd* and 50 μL broth. We heat-killed *Bd* by incubating it at 60°C for 1 h. Each plate was prepared in triplicate, with one set incubated at each of three temperatures (14, 20, and 26°C). We read each plate daily on a Spectramax 190 spectrophotometer with a 492 nm filter for ten days. We also inspected each plate visually each day during the growth phase to record wells in which *Bd* growth inhibition had occurred and to exclude any wells in which obvious contamination had developed. Plates were kept in their respective incubators between daily readings. Extracts from all 44 visibly distinct bacterial isolates were included in this experiment.

### 2.5. Analyses

We compared the growth of *Bd* among temperature treatments and bacterial extracts using the mean optical density (OD) value on day 10 of the challenge assay, which occurred during the exponential growth phase. Wells that had visible contamination were excluded from the analyses. To compare the growth of *Bd* in the presence of bacterial extracts to positive controls (no extracts), we used a relative growth index. To calculate this index, we followed the method in Bell et al. (2013). We first subtracted the negative control from each sample then we subtracted the mean OD on Day 0 from the mean OD on Day 10 for each well containing a bacterial extract or positive control. We then divided this number by the corrected (day 10 minus day 0) OD of the positive controls to give a percentage of *Bd* growth for each well containing a bacterial extract relative to wells containing no extracts (positive controls). We then subtracted 1 from these values so that negative numbers represent the percent reduction of growth relative to the positive controls (percent inhibition) and positive numbers represent the percent of growth over and above that of the positive controls (percent growth facilitation). A value of 0 represents no difference in growth between the experimental well containing a bacterial extract and the positive *Bd* growth controls.

To assign bacterial isolates to categories of “inhibitory,” “no effect,” and “facilitating” of *Bd* growth for each challenge temperature, we used cut-off values based on proportional growth comparisons to positive *Bd* growth controls: bacterial extracts that consistently (in all 3 replicates) resulted in a ≥ 80% reduction in *Bd* growth in comparison to positive controls at a given temperature (index values ≤ −0.8) were considered inhibitory, extracts that resulted in a ≥ 20% increase in *Bd* growth in comparison to controls (index values ≥1.2) were considered facilitating, and extracts producing values in between these ranges were considered to have no effect on *Bd* growth. Wells that had index values ≥3.0, indicating growth at ≥300% of the positive control (*n* = 2), were excluded under the assumption that these harbored contamination that was missed in our initial visual screen.

We performed all statistical analyses using R Studio 2022 (Racine, 2012) with R version 2022.02.1 (R Core Team, 2019) and produced figures using ggplot2 version 3.4.3 (Wickham, 2011). We used DHARMa version 0.4.6 (Hartig and Hartig, 2017) and visual assessments of residuals plots to confirm that model assumptions were met. To test for differences in *Bd* growth index across bacterial incubation and *Bd* challenge temperatures, we used a linear mixed model (LMM: ‘nlme’ package version 3.1–163, function ‘lme’) (Pinheiro et al., 2023) with bacterial incubation temperature, *Bd* challenge assay temperature, species, and their interactions as fixed effects and plate as a random effect. We ran this model with the full set of morphologically distinct bacterial isolates as well as a reduced dataset consisting of the set of isolates that were < 99% identical in their 16 s sequences (including 1 randomly chosen isolate per group of ≥99% matching isolates).

## 3. Results

### 3.1. Sequence identification

BLAST results for the consensus sequences for each isolate are shown in Table 1. The 44 isolates that we sequenced are representatives of 13 families of bacteria (Table 1). Six of these families were found on both frog species, three were only found on *A. blanchardi*, and four were only found on *R. sphenocephala*. These 44 isolates sorted into 26 isolate groups (groups with >99% sequence similarity). Seventeen isolates with at least 1% 16 s sequence difference from one another were obtained from *A. blanchardi* and 14 were obtained from *R. sphenocephala*. Only 5 of the isolates from this shorter list were found on both frog species. The “full dataset” for subsequent statistical analysis included all 44 isolates and the “reduced” dataset included just the shorter list of 26 isolates (one chosen haphazardly from each group) that differ by >1% from all other isolate groups in their 16 s sequences.

**Table 1:** BLAST results for sequenced bacterial isolates from *A. blanchardi* (ACBL) and *R. sphenocephala* (RASP).

Isolate taxonomy						Effect on *Bd* growth in Woodhams et al. (2015)	
**Family**	**Species**	**≥ 99% 16 s groups**	**Source**	**Isolate ID**	**Effect on *Bd* growth in this study**	**100% match**	**≥ 99% match**
Alcaligenaceae	*Advenella* sp.	A	RASP	52	no effect		
Bacillaceae	*Bacillus* sp.	B	ACBL	23	no effect	inhibitory, facilitating, no effect	inhibitory, facilitating, no effect
Bacillaceae	*Bacillus* sp.	C	ACBL	17	no effect	inhibitory, facilitating, no effect	inhibitory, facilitating, no effect
Bacillaceae	*Bacillus* sp.	C	ACBL	34	inhibitory (14:26, 20:14, 20:26), no effect	inhibitory, facilitating, no effect	inhibitory, facilitating, no effect
Bacillaceae	*Bacillus* sp.	C	RASP	57	no effect	inhibitory, facilitating, no effect	inhibitory, facilitating, no effect
Bacillaceae	*Lysinibacillus* sp.	D	ACBL	32	inhibitory (14:26), no effect	inhibitory, no effect	inhibitory, no effect
Bacillaceae	*Lysinibacillus* sp.	D	RASP	72	no effect	no effect	inhibitory, no effect
Bacillaceae	*Lysinibacillus* sp.	D	RASP	73	no effect	no effect	inhibitory, no effect
Burkholderiaceae	*Paraburkholderia* sp.	E	ACBL	2	no effect		
Caulobacteraceae	*Brevundimonas* sp.	F	ACBL	3	inhibitory (20:20), no effect		inhibitory, no effect
Enterobacteriaceae	*Enterobacter* sp.	G	ACBL	5	no effect		
Enterobacteriaceae	*Enterobacter* sp.	H	RASP	58	no effect	no effect	inhibitory, facilitating, no effect
Erwiniaceae	*Pantoea* sp.	I	ACBL	10	facilitating (14:20), no effect		
Erwiniaceae	*Pantoea* sp.	J	ACBL	18	no effect		inhibitory, no effect
Erwiniaceae	*Pantoea* sp.	J	ACBL	45	inhibitory (14:14, 20:14), no effect	no effect	inhibitory, no effect
Erwiniaceae	*Pantoea* sp.	K	RASP	70	facilitating (14:26), no effect	no effect	inhibitory, no effect
Erwiniaceae	*Pantoea* sp.	L	RASP	92	no effect		inhibitory, no effect
Exiguobacteraceae	*Exiguobacterium* sp.	M	RASP	56	no effect		inhibitory, facilitating, no effect
Microbacteriaceae	*Curtobacterium* sp.	N	ACBL	25	no effect		inhibitory, facilitating, no effect
Microbacteriaceae	*Curtobacterium* sp.	N	ACBL	40	inhibitory (14:14, 14:20, 20:20), no effect	inhibitory, no effect	inhibitory, facilitating, no effect
Microbacteriaceae	*Curtobacterium* sp.	N	RASP	94	inhibitory (14:14, 14:20), no effect		inhibitory, facilitating, no effect
Microbacteriaceae	*Microbacterium* sp.	O	ACBL	12	no effect	inhibitory, no effect	inhibitory, facilitating, no effect
Microbacteriaceae	*Microbacterium* sp.	O	RASP	86	no effect	inhibitory, no effect	inhibitory, facilitating, no effect
Microbacteriaceae	*Microbacterium* sp.	P	ACBL	13	no effect	inhibitory, facilitating	inhibitory, facilitating, no effect
Microbacteriaceae	*Microbacterium* sp.	Q	ACBL	24	no effect	inhibitory, no effect	inhibitory, no effect
Micrococcaceae	*Arthrobacter* sp.	R	RASP	53	no effect		
Paenibacillaceae	*Paenibacillus* sp.	S	ACBL	8	no effect		
Weeksellaceae	*Chryseobacterium indologenes*	T	RASP	78	no effect		inhibitory, facilitating, no effect
Weeksellaceae	*Chryseobacterium* sp.	U	ACBL	1	no effect		inhibitory
Weeksellaceae	*Chryseobacterium* sp.	V	ACBL	46	no effect		inhibitory
Weeksellaceae	*Chryseobacterium* sp.	W	RASP	54	no effect		inhibitory, no effect
Weeksellaceae	*Chryseobacterium* sp.	W	RASP	61	no effect		inhibitory, no effect
Weeksellaceae	*Chryseobacterium* sp.	W	RASP	62	no effect		inhibitory, no effect
Weeksellaceae	*Chryseobacterium* sp.	W	RASP	63	no effect		inhibitory, no effect
Weeksellaceae	*Chryseobacterium* sp.	W	RASP	68	no effect		inhibitory, no effect
Weeksellaceae	*Chryseobacterium* sp.	W	RASP	82	no effect		inhibitory, no effect
Weeksellaceae	*Chryseobacterium* sp.	W	RASP	85	no effect		inhibitory, no effect
Weeksellaceae	*Chryseobacterium* sp.	W	RASP	96	no effect		inhibitory, no effect
Weeksellaceae	*Chryseobacterium* sp.	X	RASP	98	no effect		
Xanthomonadaceae	*Stenotrophomonas* sp.	Y	ACBL	30	no effect	inhibitory	inhibitory, facilitating, no effect
Xanthomonadaceae	*Stenotrophomonas* sp.	Y	ACBL	41	no effect		inhibitory, facilitating, no effect
Yersiniaceae	*Serratia marcescens*	Z	ACBL	14	no effect	inhibitory, facilitating	inhibitory, facilitating, no effect
Yersiniaceae	*Serratia marcescens*	Z	ACBL	15	no effect	inhibitory, facilitating	inhibitory, facilitating, no effect
Yersiniaceae	*Serratia marcescens*	Z	RASP	75	inhibitory (20:14, 26:14), no effect	inhibitory	inhibitory, no effect

The table shows all 44 of the morphologically distinguishable isolates with isolates with **≥**99% 16 s sequence similarity to one another indicated by letter codes in the third column. The 6^th^ column indicates whether the isolate’s extract inhibited (followed by at which bacterial incubation and *Bd* challenge temperature combination(s)), had no effect across some temperature combinations, or facilitated *Bd* growth (followed by at which bacterial incubation and *Bd* challenge temperature combination(s)) in this study, and the 7th and 8th columns indicate the same information for isolates that matched our isolates’ 16 s sequences (to 100% and ≥ 99%) and were included in the Woodhams et al. (2015) database. In these columns listing of multiple effect categories (e.g., inhibitory/facilitating) indicates that contrasting results were reported from separate studies included in the Woodhams et al. (2015) database.

### 3.2. Differences in relative growth

**Figure 1:**
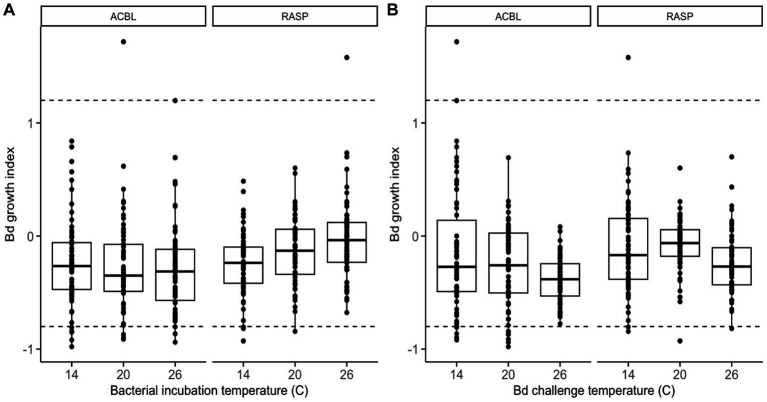
Relative growth of *Bd*, as compared to positive growth controls, when challenged to grow in the presence of frog skin bacterial extracts from two frog species (ACBL = *Acris blanchardi*, RASP = *Rana sphenocephala*) at three different temperatures. In **(A)**, the data are organized by bacterial incubation temperature and in **(B)** they are organized by *Bd* challenge temperature. Negative values indicate inhibition of *Bd* growth and positive values indicate facilitation as compared to no-extract controls. Isolates with values under −0.8 (lower dashed line) were considered inhibitive and isolates with values over 1.2 (upper dashed line) were considered facilitating of *Bd* growth.

When considering the full dataset of 44 morphologically distinct bacterial isolates, we found no significant main effect of bacterial incubation temperature on *Bd*’s ability to grow (relative growth index) in the presence of bacterial extracts (LMM: χ^2^ = 1.449, *p* = 0. 485, Supplementary Table S1). There was, however, a significant interaction between bacterial incubation temperature and frog species (LMM: χ^2^ = 8.515, *p* = 0.014). Bacterial products from *R. sphenocephala* isolates inhibited *Bd* growth more strongly when the isolates were incubated at 14°C than at 26°C (LMM: t = 2.869, *p* = 0.004; Figure 1A). The temperature at which the growth challenge assay was conducted also affected *Bd’*s relative growth index (LMM: χ^2^ = 6.996, *p* = 0.030, Figure 1B). *Bd* growth was inhibited more strongly when this growth challenge assay took place at 26°C than at 14°C (LMM: t = −2.639, *p* = 0.009, Supplementary Table S2). None of the other two- or three-way interactions in the model were significant (LMM: χ^2^ ≤ 2.657, *p* ≥ 0.237).

When considering the reduced dataset of only the set of 26 bacterial isolates with 16 s sequences that differed by >1%, we found that none of the main effects, two-, or three-way interactions in the model were significant (Supplementary Tables S3, S4 and Supplementary Figure S1). The interaction between bacterial incubation temperature and frog species was nearly significant (LMM: χ^2^ = 5.061, *p* = 0.080) and showed the same pattern as in the full dataset: bacterial products from *R. sphenocephala* isolates inhibited *Bd* growth more strongly when they had been incubated at lower temperatures.

### 3.3. Differences in categorical effects on *Bd* growth

**Figure 2:**
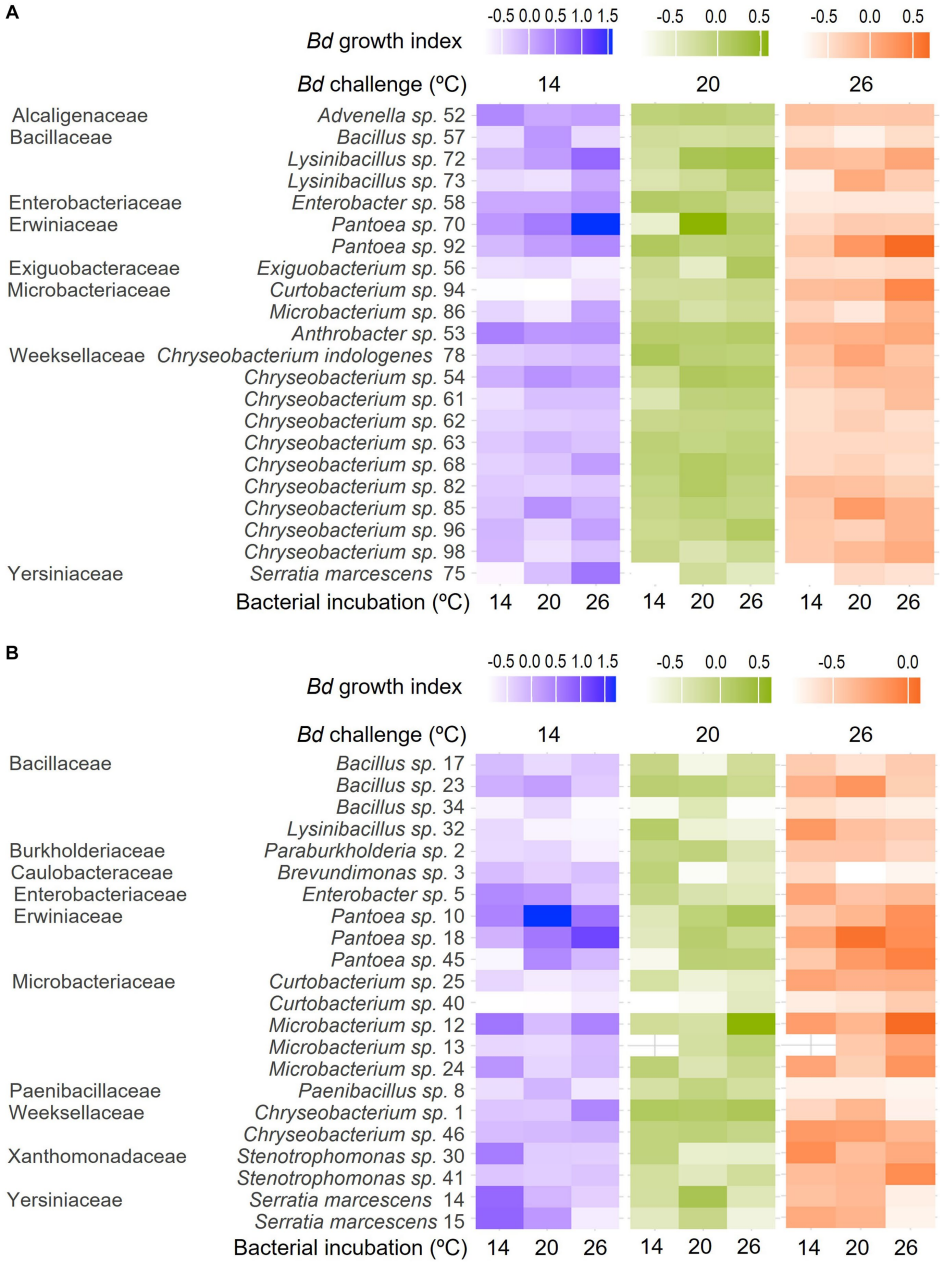
Heat maps showing differences in *Bd* growth index across bacterial incubation temperatures (individual columns) and *Bd* challenge temperatures (differently colored groups of columns) for bacteria isolated from the skin of **(A)** *R. sphenocephala* and **(B)** *A. blanchardi.* Lighter shading (lower Bd growth index) corresponds to more inhibition of the fungal pathogen’s growth in *in vitro* challenge assays. Bacterial isolates are grouped by family, genus, and isolate ID, as they also are in Table 1.

None of the bacterial isolates were inhibitive of *Bd* growth across all the bacterial incubation and growth challenge assay temperature combinations (Figure 2, Table 1). Many were inhibitive only in a single combination, but a few were inhibitive in up to three of the nine combinations tested. For example, the extract from isolate 34 (*Bacillus* sp.) was inhibitive of *Bd* growth when incubated at 14°C and challenged at 26°C and when incubated at 20°C and challenged at 14°C or 26°C but not under other combinations tested. None of the bacterial extracts were inhibitive across all three bacterial incubation temperatures. Likewise, none of the extracts produced at a given bacterial incubation temperature were inhibitive across all three *Bd* challenge temperatures.

Nineteen of the 44 bacterial isolates we isolated and sequenced were a 100% sequence match to isolates in the Woodhams et al. (2015) dataset (version updated in 2020 by MC Bletz and DC Woodhams). Our categorical findings (inhibitory, facilitating, or no effect) for effects on *Bd* of extracts from these isolates matched with those in the database for all but six of the isolates where either we found no effect on *Bd* growth and the Woodhams et al. dataset showed evidence of inhibition (4 isolates) or we found evidence of facilitation (1 isolate) or inhibition (1 isolate) but the studies in the Woodhams et al. dataset found no effect. Another 18 isolates from this study were a > 99% but <100% sequence match to the Woodhams et al. dataset. For 16 of these we found no effect of their extract on *Bd* growth and for two we found evidence *Bd* inhibition under some conditions. These categorical findings agreed with those in the Woodhams et al. dataset in all but three cases where either we found no effect and the Woodhams et al. dataset contained evidence of inhibition (2 isolates) or where we found facilitation and the Woodhams et al. dataset did not (1 isolate). Seven of the isolates we identified were not found in the Woodhams et al. database (< 99% sequence match) at all. For six of these we found no effect on *Bd* growth and for one we found facilitation of *Bd* growth.

## 4. Discussion

Thirteen of the 15 genera and all 13 families of bacteria identified in this study have been previously found on frog skin. *Arthrobacter* (Micrococcaceae; Lauer et al., 2008), *Bacillus* (Bacillaceae; Loudon et al., 2014), *Brevundimonas* (Caulobacteraceae; Roth et al., 2013), *Chryseobacterium* (Weeksellaceae; Mauel et al., 2002), *Curtobacterium* (Microbacteriaceae; Becker et al., 2015a,b), *Enterobacter* (Enterobacteriaceae; Pasteris et al., 2009), *Exiguobacterium* (Exiguobacteraceae; Xu et al., 2020), *Lysinibacillus* (Bacillaceae; Kueng et al., 2014), *Microbacterium* (Microbacteriaceae; Suzina et al., 2015), *Paenibacillus* (Paenibacillaceae; Susilawati et al., 2021), *Pantoea* (Erwiniaceae; Brito de Assis et al., 2016), *Serratia* (Yersiniaceae; Bresciano et al., 2015), and *Stenotrophomonas* (Xanthomonadaceae; O’Connell et al., 2011) have all been reported from frog skin, though in some cases ours is the first report from North American frogs. *Advenella* (Alcaligenaceae) and *Paraburkholderia* (Burkholderiaceae) were found in this study but to our knowledge these genera not been reported from frog skin previously. However, *Pigmentiphaga*, another genus in the Alcaligenaceae, has been found on frog skin, specifically in association with arboreal frogs (Bletz et al., 2019). Four of the identified bacterial families were found on *A. blanchardi* only, three were found on *R. sphenocephala* only, and six were found on both host species (Table 1).

All 13 of the bacterial families and all 13 of the previously reported genera from this study have members that have been found before, at least under some conditions, to inhibit the growth of *Bd in vitro* (Woodhams et al., 2015). However, for many of the bacteria we identified the results in the Woodhams et al. (2015) dataset were mixed, suggesting that in some studies their products were found to inhibit *Bd* growth whereas in other studies they were not. One potential reason for this variation could have been differences in bacterial growth media among the included studies. It stands to reason that bacteria would produce different metabolites when grown on different food sources and this could have impacted the results of *Bd* growth challenge assays using cell free supernatants. Differences in the *Bd* strain used and/or its history in culture may have also contributed to the variation seen among the studies in the Woodhams et al. (2015) dataset. Another source of variation could have been differences in the temperatures at which *in vitro* growth challenge assays were done across the many studies in the Woodhams et al. (2015) dataset. These previous studies ranged in temperature from 15 to 26°C but the vast majority were done at “room temperature,” which was defined as 21 to 23°C. For the two new genera we report here (*Advenella* and *Paraburkholderia*) we did not find evidence of *Bd* inhibition at any temperature. However, for many of the previously reported bacteria we also isolated, despite the fact that we used a consistent bacterial growth medium and *Bd* strain, we found that whether a bacterial isolate’s extracts facilitated, inhibited, or had no effect on *Bd* differed among our temperature treatments. In fact, none of the isolates from this study inhibited *Bd* growth across all the temperature treatments we tried, and most were inhibitive at only one or a few of temperature treatments.

Although temperature has been found to affect *Bd* growth both in culture and on amphibian skin, this study was the first to consider the effects of temperature on both the production of anti-*Bd* metabolites by cutaneous bacteria *and* the ability of those metabolites to inhibit *Bd* growth. Standardizing the optical density (OD) of bacteria and their products used in the growth challenge assay also permitted us to disentangle effects of temperature on bacterial growth from effects on the anti-fungal properties of bacterial supernatants. We predicted that the bacteria we isolated from amphibian skin would produce more metabolites with anti-*Bd* properties near their own optimal growth temperatures, which we did not measure but predicted would be similar to the typical body temperatures of their hosts (mean of 19.5°C for *R. sphenocephala* and 22.5°C for *A. blanchardi*). Thus, we predicted that *Bd* inhibition would be greater at lower bacterial incubation temperatures for isolates collected from *R. sphenocephala* than for those collected from *A. blanchardi*. We also predicted that antifungal metabolites produced by these bacteria would be more effective at inhibiting *Bd* growth at temperatures that are suboptimal for *Bd* growth *in vitro,* i.e., at temperatures away from 23°C, which is considered optimal for growth of *Bd* in culture (Piotrowski et al., 2004). Thus, we predicted that *Bd* growth inhibition would be greater at 14 and 26 than at 23°C.

The pattern we observed among the bacterial incubation temperatures does support the prediction that *R. sphenocephala* isolates are better at inhibiting *Bd* growth when incubated at lower temperatures. However, *Bd* growth was lowest when these isolates were incubated at 14°C whereas the mean body temperature for *R. sphenocephala* in the southern part of its range, where our collections took place, is closer to our 20°C treatment temperature. There was also no clear pattern with bacterial incubation temperature for isolates collected from *A. blanchardi*. Taken together, these data suggest that the temperature at which bacterial isolates are grown can, in fact, influence the ability of their metabolites to inhibit *Bd* growth, though the temperature at which metabolites are most effective may not be closely matched to the thermal ecology of the amphibian host species. It stands to reason that the thermal physiology of the bacteria themselves is an important driver of variation in metabolite production with temperature. However, predicting which temperatures may be optimal for antifungal metabolite production given the suite of bacteria that produce them on amphibian skin, may be beyond our reach without a more detailed understanding of the biology of these bacterial taxa. Future work characterizing differences in metabolites produced across culture temperatures and among bacterial taxa is needed to clarify the mechanisms generating temperature-driven differences in the antimicrobial properties of symbiotic microbes in general, and in frog skin microbes in particular.

*Bd* has been shown to grow in culture between the temperatures of 4°C and 28°C, but the optimal range for growth *in vitro* is between 17°C and 25°C (Piotrowski et al., 2004). Our 14°C and 26°C challenge temperature treatments were chosen to be within the range for *Bd* growth, but outside the optimal growth range. Our second prediction, that *Bd* growth would be inhibited more by bacterial metabolites when challenged away from its optimal growth temperature, was partially supported. *Bd* growth index was lower when the challenge temperature was 26°C, a temperature near the upper limit for growth of this pathogen. However, we did not see the same pattern below the optimal growth temperature range. There was no difference in *Bd* growth index between challenge temperatures of 14 and 20°C. An explanation for this pattern may come from the asymmetrical nature of thermal performance curves. Across many taxa, these curves generally have a steeper decline from the thermal optimum to the critical thermal maximum (CT_max_) than they do from the thermal optimum to the critical thermal minimum (CT_min_), giving them a “left skewed” appearance (Schulte et al., 2011). As thermal performance curves describe the effects of temperature on biological rate processes, including processes like growth, fecundity, metabolic rate and enzyme activity, it stands to reason that Bd growth might be more easily inhibited by bacterial metabolites at temperatures near CT_max_, where these processes begin to fail. Our lower temperature of 14°C is still quite far from Bd’s CT_min_, which could explain the lack of a difference in Bd growth index between challenge assays done at this temperature vs. 20°C, which is near the thermal optimum. Given that both amphibians and their *Batrachochytrium* pathogens experience a range of environmental conditions in the wild, insights like these, that help to clarify our understanding of the impacts of temperature on mechanisms of host defense, are needed to predict susceptibility to chytridiomycosis.

Because we were interested in knowing whether the members of the amphibian skin microbiome produce their antifungal metabolites better or faster at certain temperatures, we chose to grow all bacterial isolates to a set optical density (OD) before removing bacteria and using the supernatants in our *Bd* growth challenge assays. Our results do, in fact, suggest that temperature can alter the antifungal properties of bacterial supernatants independent of their density in culture, as we saw an effect of bacterial incubation temperature on Bd growth inhibition in the skin microbes from *R. sphenocephala*. Another approach, and the one taken by Daskin et al. (2014), would have been to grow each bacterial isolate for sufficient time to see a plateau in OD before harvesting extracts for *Bd* growth challenge assays. With this approach Daskin et al. (2014) also found an effect of incubation temperature on the ability of bacterial extracts to inhibit *Bd* growth though their findings went in a different direction than our own. Focusing just on isolates that were known to be *Bd*-inhibitory, they found that *Bd* growth inhibition by bacterial metabolites was reduced at low temperatures whereas our findings show a trend in the opposite direction. Because the bacteria in Daskin et al. (2014) were not grown to a common density, the differences they found with temperature could stem from differences in bacterial densities more so than differences in the types or quantities of antifungal metabolites produced across incubation temperatures. In other words, the metabolite cocktails from cultures grown under lower temperatures may have been less effective at inhibiting the growth of *Bd* simply because at low temperatures there were fewer bacteria present to produce those metabolites. This key methodological difference could explain the difference in results between the Daskin et al. (2014) study and our own. An alternative explanation is that the antifungal activity of bacterial communities on the Australian rainforest treefrogs sampled by Daskin et al. (2014) shows a different relationship with temperature than that of the bacterial communities of the subtropical North American pond frogs sampled in this study. Future work comparing the effects of temperature on the antimicrobial properties of amphibian skin microbes across a greater range of host ecologies is needed to tease apart these alternatives.

Our use of 1% tryptone broth, a growth environment that is undoubtedly quite different from amphibian skin, may also have affected what metabolites were produced by the bacterial isolates in this study. Valerio et al. (2016) found that manipulating the growth medium of lactic acid bacteria by adding phenylpyruvic acid improved their antifungal activity. The bacteria utilized in our study may have been able to produce more or different antifungal compounds across temperatures in other growth environments, for example while on the skin of frogs, but not when grown on low nutrient agar. Because our growth challenge assay used only bacterial supernatants, this study also did not address whether and how quickly amphibian skin bacteria would produce antifungal metabolites when grown in the presence of *Bd* and how that might vary with temperature.

Although it is generally accepted that temperature plays a large role in the dynamics of chytridiomycosis infections, this study helps to clarify some of the mechanisms that may be driving this relationship. While amphibian immune systems in general are thought to function more effectively against *Bd* at higher temperatures (Rollins-Smith et al., 2011), our findings, the Woodhams et al. (2015) database, and the Daskin et al. (2014) study all suggest that temperature also modulates anti-fungal properties of amphibian skin bacterial communities, which are an important first-line defense against *Bd* and other skin pathogens. While among the many studies in the Woodhams et al. (2015) database there could be many sources of variation, our results and the results of Daskin et al. (2014) suggest that this variation could be due to differences in bacterial titer or density, the production of different metabolites across temperatures, or possibly both of these factors. A similar effect on bacterial titer to that shown in Daskin et al. (2014) has been seen to affect the benefits that inherited microbial symbionts provide to their hosts (rev. in Corbin et al., 2017). If there is a minimum concentration of metabolites necessary to inhibit *Bd* growth, it seems plausible that *Bd*-inhibitory bacteria will produce the necessary amount of metabolites faster when grown at their own optimal temperature than when grown below or above that temperature. However, our understanding of optimal temperatures for growth and antifungal metabolite production in amphibian skin microbes, and in fact in symbiotic bacteria more broadly, remains in its infancy. Filling this gap in our understanding of microbial interactions will be necessary in order to predict the effects of changes in temperature, both natural and anthropogenic, on the symbiotic relationships that animals depend on for a range of functions, from digestion to protection from pathogens.

## Data availability statement

Sequence data generated for this project is available on GenBank (Accession Nos. OR228691 - OR228864). The raw data supporting the conclusions of this article will be made available by the authors, without undue reservation.

## Ethics statement

The animal study was approved by Tulane University Institutional Animal Care and Use Committee (IACUC). The study was conducted in accordance with the local legislation and institutional requirements.

## Author contributions

MR: Conceptualization, Data curation, Investigation, Methodology, Writing – original draft, Writing – review & editing. VS: Formal analysis, Validation, Visualization, Writing – review & editing. EC: Formal analysis, Visualization, Writing – review & editing. CR-Z: Conceptualization, Data curation, Formal analysis, Funding acquisition, Investigation, Methodology, Project administration, Resources, Supervision, Validation, Visualization, Writing – review & editing.
