# Supplementary material for: Effects of temperature on the interaction between amphibian skin bacteria and Batrachochytrium dendrobatidis
Source: Front Microbiol. 2023 Oct 24;14:1253482. doi: 10.3389/fmicb.2023.1253482 (PMC10628663; doi:10.3389/fmicb.2023.1253482)
Supplement: Supplementary file 1 [file Data_Sheet_1.docx]

**SUPPLEMENTAL MATERIAL**

**Effects of temperature on the interaction between amphibian skin bacteria and *Batrachochytrium dendrobatidis***

Matthew J. Robak, Veronica Saenz, Esmee de Cortie, and Corinne L. Richards-Zawacki

SUPPLEMENTAL FIGURES


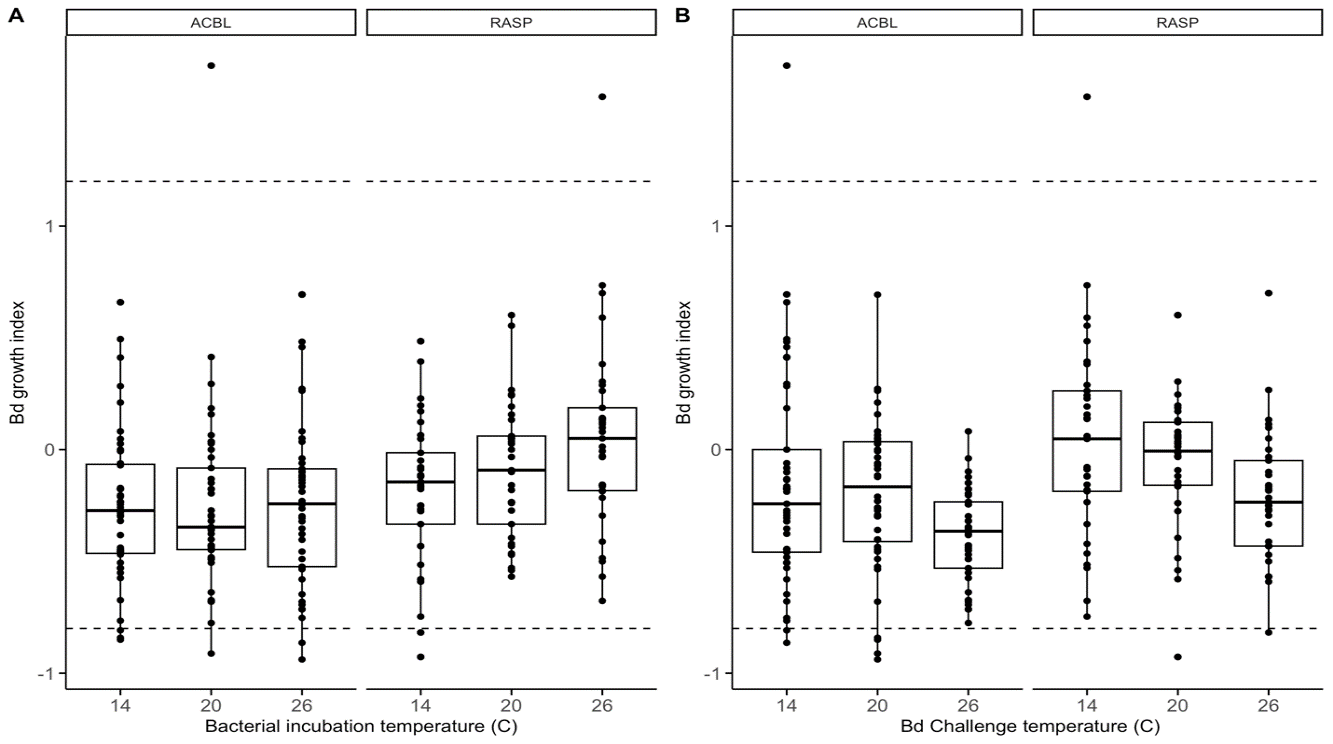


**Figure 1.** Relative growth of *Bd* for the set of 26 bacterial isolates with < 99% 16s sequence similarity to one another, as compared to positive growth controls, when challenged to grow in the presence of frog skin bacterial extracts from two frog species (ACBL = *Acris blanchardi*, RASP = *Rana sphenocephala*). In (A), the data are organized by bacterial incubation temperature and in (B) they are organized by *Bd* challenge temperature. Negative values indicate inhibition of *Bd* growth and positive values indicate enhancement as compared to no-extract controls. Bacterial isolates producing *Bd* growth index values < -0.8 (below dashed line) were considered “inhibitive” and those producing values > 1.2 were considered “facilitating” of *Bd* growth.

STATISTICAL MODEL OUTPUTS

**Table S1.** Output (ANOVA table) from a linear mixed model of *Bd* growth index across the three challenge temperatures and three bacterial growth temperatures in the two frog species.

|  | Chi-squared | DF | P-value |
| --- | --- | --- | --- |
| Intercept | 1.521 | 1 | 0.218 |
| *Bd* challenge temperature | **6.996** | **2** | **0.030** |
| Bacterial incubation temperature | 1.449 | 2 | 0.485 |
| Frog species | 1.401 | 1 | 0.237 |
| *Bd* challenge temp. x bacterial incubation temp. | 0.814 | 4 | 0.936 |
| *Bd* challenge temp. x frog species | 2.435 | 2 | 0.296 |
| Bacterial incubation temp. x frog species | **8.515** | **2** | **0.014** |
| *Bd* incubation temp. x bacterial incubation temp. x frog species | 2.657 | 4 | 0.617 |

**Table S2.** Output from the same linear mixed model of *Bd* growth index across the three challenge temperatures and three bacterial incubation temperatures in the two frog species as in the ANOVA table above. Coefficient estimates and their standard errors (in parentheses) are shown for each factor.

|  | | |  |
| --- | --- | --- | --- |
|  | Dependent variable: |  |  |
|  |  |  |  |
|  | *Bd* growth index |  |  |
|  | | | |
| *Bd* challenge temperature = 20 °C | -0.155 (-0.365, 0.06) |  |  |
|  | t = -1.447 |  |  |
|  | p = 0.149 |  |  |
| *Bd* challenge temperature = 26 °C | -0.283^**^ (-0.492, -0.073) |  |  |
|  | t = -2.639 |  |  |
|  | **p = 0.009** |  |  |
| Bacterial incubation temperature = 20 °C | -0.051 (-0.258, 0.157) |  |  |
|  | t = -0.479 |  |  |
|  | p = 0.632 |  |  |
| Bacterial incubation temperature = 26 °C | -0.127 (-0.334, 0.081) |  |  |
|  | t = -1.196 |  |  |
|  | p = 0.233 |  |  |
| Species (*R. Sphenocephala*) | -0.128 (-0.340, 0.084) |  |  |
|  | t = -1.184 |  |  |
|  | p = 0.238 |  |  |
| *Bd* challenge temperature = 20 °C x bacterial incubation temperature = 20 °C | 0.014 (-0.281, 0.309) |  |  |
|  | t = 0.094 |  |  |
|  | p = 0.925 |  |  |
| *Bd* challenge temperature = 26 °C x bacterial incubation temperature = 20 °C | -0.024 (-0.271, 0.319) |  |  |
|  | t = 0.162 |  |  |
|  | p = 0.871 |  |  |
| *Bd* challenge temperature = 20 °C x bacterial incubation temperature = 26 °C | 0.110 (-0.185, 0.405) |  |  |
|  | t = 0.733 |  |  |
|  | p = 0.464 |  |  |
| *Bd* challenge temperature = 26 °C x bacterial incubation temperature = 26 °C | 0.108 (-0.187, 0.405) |  |  |
|  | t = 0.719 |  |  |
|  | p = 0.472 |  |  |
| *Bd* challenge temperature = 20 °C x frog species (*R. Sphenocephala*) | 0.226 (-0.069, 0.521) |  |  |
|  | t = 1.500 |  |  |
|  | p = 0.134 |  |  |
| *Bd* challenge temperature = 26 °C x frog species (*R. Sphenocephala*) | 0.168 (-0.127, 0.463) |  |  |
|  | t = 1.114 |  |  |
|  | p = 0.266 |  |  |
| Bacterial incubation temperature = 20 °C x frog species (*R. Sphenocephala*) | 0.146 (-0.148, 0.439) |  |  |
|  | t = 0.974 |  |  |
|  | p = 0.331 |  |  |
| Bacterial incubation temperature = 26 °C x frog species (*R. Sphenocephala*) | 0.429** (0.136, 0.733) |  |  |
|  | t = 2.869 |  |  |
|  | **p = 0.004** |  |  |
| *Bd* challenge temperature = 20 °C x bacterial incubation temperature = 20 °C x frog species (*R. Sphenocephala*) | 0.003 (-0.413, 0.419) |  |  |
|  | t = 0.016 |  |  |
|  | p = 0.988 |  |  |
| *Bd* challenge temperature = 26 °C x bacterial incubation temperature = 20 °C x frog species (*R. Sphenocephala*) | 0.001 (-0.416, 0.416) |  |  |
|  | t = 0.002 |  |  |
|  | p = 0.998 |  |  |
| *Bd* challenge temperature = 20 °C x bacterial incubation temperature = 26 °C x frog species (*R. Sphenocephala*) | -0.280 (-0.696, 0.136) |  |  |
|  | t = -1.319 |  |  |
|  | p = 0.188 |  |  |
| *Bd* challenge temperature = 26 °C x bacterial incubation temperature = 26 °C x frog species (*R. Sphenocephala*) | -0.227 (-0.643, 0.189) |  |  |
|  | t = -1.071 |  |  |
|  | p = 0.285 |  |  |
| Constant | -0.09 (-0.246, 0.056) |  |  |
|  | t = -1.233 |  |  |
|  | p = 0.219 |  |  |
|  | | | |
| Observations | 394 |  |  |
| Log Likelihood | -168.306 |  |  |
| Akaike Inf. Crit. | 376. 611 |  |  |
| Bayesian Inf. Crit. | 455.203 |  |  |
|  | | | |
| Note: | ^*^p^**^p^***^p<0.001 |  |  |

**Table S3.** Output (ANOVA table) from a linear mixed model of *Bd* growth index across the three challenge temperatures and three bacterial incubation temperatures in the two frog species using only the set of 26 bacterial isolates with < 99% 16s sequence similarity to one another.

|  | Chi-squared | DF | P-value |
| --- | --- | --- | --- |
| Intercept | 3.366 | 1 | 0.067 |
| *Bd* challenge temperature | 2.956 | 2 | 0.228 |
| Bacterial incubation temperature | 0.573 | 2 | 0.751 |
| Frog species | 0.437 | 1 | 0.508 |
| *Bd* challenge temp. x bacterial incubation temp. | 0.977 | 4 | 0.913 |
| *Bd* challenge temp. x frog species | 0.032 | 2 | 0.984 |
| Bacterial incubation temp. x frog species | 5.157 | 2 | 0.076 |
| *Bd* incubation temp. x bacterial incubation temp. x frog species | 3.166 | 4 | 0.531 |

**Table S4.** Output from the same linear mixed model of *Bd* growth index across the three challenge temperatures and three bacterial incubation temperatures in the two frog species as in the ANOVA table above using only the set of 26 bacterial isolates with < 99% 16s sequence similarity to one another. Coefficient estimates and their standard errors (in parentheses) are shown for each factor.

|  | |
| --- | --- |
|  | Dependent variable: |
|  |  |
|  | *Bd* growth index |
|  | |
| *Bd* challenge temperature = 20 °C | -0.039 (-0.293, 0.245) |
|  | t = -0.291 |
|  | p = 0.771 |
| *Bd* challenge temperature = 26 °C | -0.219 (-0.472, 0.066) |
|  | t = -1.624 |
|  | p = 0.106 |
| Bacterial incubation temperature = 20 °C | 0.081 (-0.168, 0.361) |
|  | t = 0.614 |
|  | p = 0.540 |
| Bacterial incubation temperature = 26 °C | -0.009 (-0.259, 0.270) |
|  | t = -0.076 |
|  | p = 0.940 |
| Species (*Rana Sphenocephala*) | 0.096 (-0.201, 0.400) |
|  | t = 0.661 |
|  | p = 0.510 |
| *Bd* challenge temperature = 20 °C x bacterial incubation temperature = 20 °C | -0.108 (-0.497, 0.251) |
|  | t = -0.570 |
|  | p = 0.569 |
| *Bd* challenge temperature = 26 °C x bacterial incubation temperature = 20 °C | -0.099 (-0.488, 0.260) |
|  | t = -0.522 |
|  | p = 0.602 |
| *Bd* challenge temperature = 20 °C x bacterial incubation temperature = 26 °C | 0.064(-0.326, 0.422) |
|  | t = 0.337 |
|  | p = 0.736 |
| *Bd* challenge temperature = 26 °C x bacterial incubation temperature = 26 °C | 0.039 (-0.351, 0.397) |
|  | t = 0.206 |
|  | p = 0.837 |
| *Bd* challenge temperature = 20 °C x frog species (*Rana Sphenocephala*) | -0.035 (-0449, 0.373) |
|  | t = -0.172 |
|  | p = 0.864 |
| *Bd* challenge temperature = 26 °C x frog species (*Rana Sphenocephala*) | -0.027 (-0.441, 0.382) |
|  | t = -0.132 |
|  | p = 0.896 |
| Bacterial incubation temperature = 20 °C x frog species (*Rana Sphenocephala*) | -0.057 (-0.468, 0.349) |
|  | t = -0.280 |
|  | p = 0.780 |
| Bacterial incubation temperature = 26 °C x frog species (*Rana Sphenocephala*) | 0.368 (-0.042, 0.774) |
|  | t = 1.812 |
|  | p = 0.071 |
| *Bd* challenge temperature = 20 °C x bacterial incubation temperature = 20 °C x frog species (*Rana Sphenocephala*) | 0.209 (-0.362, 0.785) |
|  | t = 0.724 |
|  | p = 0.470 |
| *Bd* challenge temperature = 26 °C x bacterial incubation temperature = 20 °C x frog species (*Rana Sphenocephala*) | 0.199 (-0.372, 0.775) |
|  | t = 0.690 |
|  | p = 0.491 |
| *Bd* challenge temperature = 20 °C x bacterial incubation temperature = 26 °C x frog species (*Rana Sphenocephala*) | -0.271 (-0.842, 0.305) |
|  | t = -0.941 |
|  | p = 0.348 |
| *Bd* challenge temperature = 26 °C x bacterial incubation temperature = 26 °C x frog species (*Rana Sphenocephala*) | -0.188 (-0.759, 0.388) |
|  | t = -0.652 |
|  | p = 0.515 |
| Constant | -0.176 (-0.389, 0.004) |
|  | t = -1.835 |
|  | p = 0.070 |
|  | |
| Observations | 232 |
| Log Likelihood | -109.621 |
| Akaike Inf. Crit. | 259.242 |
| Bayesian Inf. Crit. | 326.374 |
|  | |
| Note: | ^*^p<0.05; ^**^p<0.01; ^***^p<0.001 |
